# The psychiatric phenotypes of 1q21 distal deletion and duplication

**DOI:** 10.1038/s41398-021-01226-9

**Published:** 2021-02-04

**Authors:** Stefanie C. Linden, Cameron J. Watson, Jacqueline Smith, Samuel J. R. A. Chawner, Thomas M. Lancaster, Ffion Evans, Nigel Williams, David Skuse, F. Lucy Raymond, Jeremy Hall, Michael J. Owen, David E. J. Linden, LeeAnne Green-Snyder, Wendy K. Chung, Anne M. Maillard, Sébastien Jacquemont, Marianne B. M. van den Bree

**Affiliations:** 1 Department of Health, Ethics and Society, Care and Public Health Research Institute (CAPHRI), Faculty of Health, Medicine and Life Sciences, Maastricht University, Maastricht, The Netherlands; 2 Division of Psychological Medicine and Clinical Neurosciences, Medical Research Council Centre for Neuropsychiatric Genetics and Genomics, Cardiff University, Cardiff, UK; 3 Preventive Neurology Unit, Wolfson Institute of Preventive Medicine, Queen Mary University of London, London, UK; 4 School of Psychology, University of Bath, Bath, UK; 5 Behavioural and Brain Sciences Unit Institute of Child Health, University College London, London, UK; 6 Cambridge Institute for Medical Research, University of Cambridge, Cambridge, UK; 7 Department of Psychiatry and Neuropsychology, School for Mental Health and Neuroscience, Faculty of Health, Medicine and Live Sciences, Maastricht University, Maastricht, The Netherlands; 8 Simons Foundation, New York, NY USA; 9 Departments of Pediatrics and Medicine, Columbia University, New York, NY USA; 10 Service des Troubles du Spectre de l’Autisme et apparentés, Centre Hospitalier Universitaire Vaudois, University of Lausanne, Lausanne, Switzerland; 11 Service de Génétique Médicale, Centre Hospitalier Universitaire Vaudois, Lausanne, Switzerland

**Keywords:** Clinical genetics, Psychiatric disorders

## Abstract

Copy number variants are amongst the most highly penetrant risk factors for psychopathology and neurodevelopmental deficits, but little information about the detailed clinical phenotype associated with particular variants is available. We present the largest study of the microdeletion and -duplication at the distal 1q21 locus, which has been associated with schizophrenia and intellectual disability, in order to investigate the range of psychiatric phenotypes. Clinical and cognitive data from 68 deletion and 55 duplication carriers were analysed with logistic regression analysis to compare frequencies of mental disorders between carrier groups and controls, and linear mixed models to compare quantitative phenotypes. Both children and adults with copy number variants at 1q21 had high frequencies of psychopathology. In the children, neurodevelopmental disorders were most prominent (56% for deletion, 68% for duplication carriers). Adults had increased prevalence of mood (35% for deletion [OR = 6.6 (95% CI: 1.4–40.1)], 55% for duplication carriers [8.3 (1.4–55.5)]) and anxiety disorders (24% [1.8 (0.4–8.4)] and 55% [10.0 (1.9–71.2)]). The adult group, which included mainly genetically affected parents of probands, had an IQ in the normal range. These results confirm high prevalence of neurodevelopmental disorders associated with CNVs at 1q21 but also reveal high prevalence of mood and anxiety disorders in a high-functioning adult group with these CNVs. Because carriers of neurodevelopmental CNVs who show relevant psychopathology but no major cognitive impairment are not currently routinely receiving clinical genetic services widening of genetic testing in psychiatry may be considered.

## Introduction

The microdeletion and the microduplication at 1q21 (chr1: 146.57–147.39; GRCh37/hg19) are enriched in patients with schizophrenia (OR estimate for the deletion: 5.2; for the duplication: 2.9)^1^ and in patients with intellectual disabilities (ID)/developmental delay (DD) and autism spectrum disorder (ASD) (OR estimate for the deletion: 35; for the duplication: 18)^2,3^. The estimates for the population frequency of the microdeletion and -duplication at this locus derived from UK Biobank are 0.027% and 0.044%, respectively^4^ although this may underestimate the true frequency in the population because more severely affected carriers are likely to be underrepresented in a middle-aged research cohort.

The clinical phenotype of 1q21 deletion^5,6^ and duplication^6^ was originally established in clinical cohorts with intellectual disability, autism or congenital anomalies.

A systematic review of 22 studies containing data from 59 children and 48 adults with the 1q21.1 duplication^7^ reported high frequencies of neurodevelopmental problems including 50.8% for DD/ID, 35.6% for autism or autistic features and 8.5% for seizures. In 2015, the Simons VIP Consortium published a comparison of 19 deletion carriers and 19 duplication carriers with familial noncarrier controls^8^. Amongst deletion carriers, the most prevalent neuropsychiatric features were mood and anxiety disorders (26%) and seizures (18%), whereas the predominant neuropsychiatric disorders in duplication carriers were ASD (41%) and ADHD (29%). A major limitation of the extant literature is that most studies have had small sample sizes and few reports have included detailed psychiatric phenotyping, and there is very little detailed clinical data from adults. In the present multi-centre study, we tripled the sample size compared to the Bernier et al.^8^ study (123 compared to 38 CNV carriers, also included in the present study). The aim of the current study was to analyse psychopathology in the largest international cohort so far assembled that comprises carriers of the reciprocal microdeletion and –duplication at 1q21 across children and adults. We specifically wanted to ascertain whether mirror phenotypes—similar to the established dose effect on head size^6^—also occur in the psychological domain and whether—and what type of—psychopathology was prevalent in an adult group largely composed of family carriers and thus not affected by the ascertainment bias of clinical samples.

## Patients and methods

Participants were recruited by three research groups/consortia: the Simons Variation in Individuals Project (VIP) consortium (*n* = 51); the CNV Research Group, Lausanne University Hospital, Lausanne, Switzerland (*n* = 12); and the Neuroscience and Mental Health Research Institute, Cardiff University, UK (*n* = 60). Unaffected family members were recruited as control participants (Simons VIP: *n* = 51; Lausanne: *n* = 10; Cardiff: *n* = 9).

### Study sample

Participants assessed in Cardiff were recruited by three projects; children by the ‘Experiences of CHildren with cOpy number Variants’ (ECHO) and ‘Intellectual Disability and Mental Health: Assessing the Genomic Impact on Neurodevelopment’ (IMAGINE-ID, http://imagine-id.org/)^9^ studies, and adults through the ‘Defining Endophenotypes From Integrated Neurosciences’ (DEFINE) study. Participants were given details of our study following a diagnosis of a 1q21.1 CNV at one of the UK National Health Service genetics clinics. The study was also advertised on support websites and social media groups for carriers of 1q21.1 CNVs. Adult participants were screened for the capacity to consent using a telephone-based protocol. If they were deemed to lack capacity, a personal consultee was contacted. All participants, or their personal consultee, provided informed written consent. For participants under the age of 16, parent/guardian consent and participant assent were obtained. For 16–18-year-old participants consent was obtained from participant and parent/guardian. All interviews were taped and decisions on psychiatric symptoms and diagnosis were made during consensus meetings led by a psychiatrist. The South-East Wales Research Ethics Committee approved the recruitment and assessment protocols used in this study (14/WA/0035). For the IMAGINE-ID study, protocols were approved by the NHS London Queen Square research ethics committee (14/LO/1069).

Participants in the Simons VIP consortium were ascertained clinically and recruited online and through clinical laboratory referrals in the United States. Data were collected at three participating U.S. sites. Further details on the methods are available in Simons VIP, 2012^10^. Participants with known additional clinically recognized CNVs were excluded. Assessments were standardized across sites through ongoing training and inter-rater reliability checks. The study was approved by the Institutional Review Boards at Columbia University (AAAF3927), Geisinger Health System (2011-0320), Children’s Hospital of Boston (12-009720), Baylor College of Medicine (H-27549) and the University of Washington (39149), and all participants 18 or older or the participant’s designated legal guardian provided written informed consent (and children under 18 were assented, when appropriate, based upon mental capacity).

Participants from the CNV Research Group (Lausanne) were taking part in a larger research project on CNVs at the 1q21.1 locus. Proband carriers were referred to the study by clinical geneticists. The study was reviewed and approved by the local ethics committee (CER-VD; PB_2016-02137) and written informed consent was obtained from participants or legal representatives before investigation. Participants were assessed at the Lausanne University Hospital, Switzerland.

### Cohort demographics

Data from 123 participants, comprising 68 deletion and 55 duplication carriers, were analysed for this study and compared with data from 70 familial controls. Participants were grouped and analysed according to carrier status (deletion, duplication and control) and age group (child < 18 years, adult ≥18 years) to avoid biasing against psychopathologies which are more prevalent at specific life-stages. For full details of participant demographics see Suppl. Table S1. No significant age or gender differences were observed between deletion carriers, duplication carriers and controls either in the child or adult cohort (Suppl. Tables S1 and S2).

Carrier status for the 1q21.1 deletion or duplication was confirmed through clinical chromosome microarrays and/or medical records. We only included carriers with a deletion or duplication that spanned at least 80% of the typical distal locus (chr1:146,527,987–147,394,444 (hg19)), but not the nearby TAR locus (Suppl. Fig. S1 and S2), based on information from clinical genetics reports or in-house genotyping. Of the 123 CNV carriers, the inheritance status was known for 86 participants (70%). Seventy-three of these individuals had a ‘known inherited’ status (85%, with one parent with a confirmed 1q21.1 CNV carrier status), 13 individuals (15%) had a ‘known de-novo’ status (both parents with negative genetic tests for a 1q21.1 CNV).

### Psychopathology and neurodevelopmental function

To assess general psychopathology in children (diagnosis as well as symptomatology), the Cardiff group used the Child and Adolescent Psychiatric Assessment (CAPA)^11^. Autism symptomatology was measured by the Social Communication Questionnaire (SCQ)^12^. The Strengths and Difficulties Questionnaire^13^ was used to establish conduct problems, emotional problems, hyperactivity problems, peer problems and prosocial functioning as well as a total SDQ score. Motor functioning was measured by the Developmental Coordination Disorder Questionnaire (DCDQ)^14^. Full methodological details have already been reported elsewhere^9^.

Adult psychopathology was assessed using the Psychiatric Assessment Schedule for Adults with Developmental Disabilities (PAS-ADD)^15^. Psychotic symptoms were assessed using the Structured Interview for Prodromal Symptoms (SIPS)^16^. Adult participants who displayed psychotic symptoms on the SIPS were further assessed using the Scale for the Assessment of Positive and Negative Symptoms (SAPS) and (SANS), respectively^17^. Adult participants in the Cardiff cohort also completed the SCID-II, a semi-structured interview for making DSM Axis II: Personality Disorder diagnoses. However, in this paper we are not reporting frequencies of personality disorders because this information was not available in the other cohorts. All diagnoses were confirmed by a licensed psychiatrist based on face-to-face interviews or review of the interview protocols. If the PAS-ADD showed evidence of autistic symptoms, the DISCO, a semi-structured interview, or the “Royal College of Psychiatrists Diagnostic Interview Guide for the Assessment of Adults with Autism Spectrum Disorder” was used to confirm the diagnosis^18,19^.

The Lausanne CNV Research Group used the Diagnostic Interview for Genetic Studies (DIGS)^20^. Each of the tools used corresponded to the DSM-IV/5 criteria for psychopathologies being assessed in this report. The Lausanne control participants had no psychopathological assessments and were therefore not included in the relevant comparisons. Children were assessed using the ADOS^21^.

In the Simons VIP, children were assessed using the ADOS^21^ and ADI-R^22^ (used for suspected ASD cases) and the DISC (Diagnostic Interview Schedule for Children)^23^ or DISC-YC (Young Child Edition), for children between 3 and 6^24^, and parent behaviour rating scales. Adults were assessed using the ADOS, ADI-R (used for suspected ASD cases up to age 26) and the self-report SCL-90-R^25^], followed by a clinical interview. Diagnoses of control participants were also based on the SCL-90-R (*n* = 23) or self-report (*n* = 28).

All centres also collected information about seizure history (separated into febrile and unprovoked seizures) although this information was not available for most of the controls.

### Cognitive functioning

In Cardiff, general cognitive ability was assessed by trained raters using the Wechsler Abbreviated Scale of Intelligence (WASI)^26^. For the children, data were also available for reaction time, sustained attention, spatial planning, and spatial working memory as measured by the Cambridge Neuropsychological Test Automated Battery (CANTAB)^27^, and set-shifting ability as measured by the Wisconsin Card Sorting Test (WCST)^28^. Adults recruited by the Simons VIP group were also assessed using the WASI, while children were assessed using the Differential Ability Scales (DAS) cognitive battery^29^ (2.5 years and older) or the Mullen Scales of Early Learning^30^ (all ages, dependent upon developmental level, resulting in a ratio IQ where applicable). The CNV Research Group used Wechsler intelligence testing for all participants as previously described^31^.

### Data analysis

Data analyses were conducted using SPSS v.23 (RRID: SCR_002865) (IBM Corp.) and the R Project for Statistical Computing (RRID:SCR_001905) (http://www.r-project.org/).

#### Categorical variables

The frequency of clinical phenotypes was analysed separately for children and adults. We compared the frequency of any DSM-IV/5 diagnosis between carriers and controls to test whether copy number variation at the locus is associated with a higher prevalence of psychopathology. For children, neurodevelopmental (ASD, ADHD, ID, social communication disorder and specific learning disorder), anxiety, mood, conduct, eating and substance use disorders were pooled across subcategories. We also pooled diagnostic categories for adults (anxiety, mood disorder, conduct disorder, neurodevelopmental, psychotic and substance use disorders). Using a random-effects logit model we tested for the effect of group status (e.g. Deletion vs Control, or Duplication vs Control) (independent variable) on the frequencies of psychiatric diagnoses (*Dependent Variable:* 0 = *No Diagnosis*, 1 = *Diagnosis Present*) while adjusting for age, sex (*Fixed Effects*) and FSIQ (only in children because of wider availability of measures) (*Random Effect*). Control individuals from families with a participant with either a deletion or a duplication were combined and comparisons for both CNVs were conducted with the same control sample. For one comparison (adult duplication effect on anxiety) we had to remove the covariate sex because all anxiety cases (controls and duplication) were female. Because our control sample consisted of family members of the carriers, case-control pairs from the same family are likely to be more similar because of shared genetic and environmental background variation. We could not always control for this because of collinearity (arising from the pooling of controls and imbalance of control recruitment across sites). Where it was possible, we ran analyses including family ID as a random factor in our models. We report results without family ID as default in Tables 1 and 2, but have added the findings of models including the effects of family ID in the footnotes of these Tables, where these could be reliably computed.

**Table 1:** The prevalence of psychiatric diagnoses in the children with 1q21 deletion or duplication and for child controls.

Children (*n* = 123)	Carrier status (*n*)							
	Deletion (*n* = 51)٭			Duplication (*n* = 44)٭			Family controls (*n* = 28)٭	
Psychiatric diagnosis (DSM-IV/5)	Number assessed	With diagnosis [%]	OR (95% CI) and *p*-value**	Number assessed	With diagnosis [%]	OR (95% CI) and *p*-value**	Number assessed	With diagnosis [%]
Any psychiatric diagnosis	49	30 [61.2]	7.11 (1.34–48.60), 0.03^a^	44	31 [70.5]	12.24 (2.62–74.84), 0.003	28	3 [10.7]
Anxiety disorder	49	12 [24.5]	1.40 (0.22–10.07), 0.72^b^	44	10 [22.7]	0.71 (0.11–4.69), 0.71	26	3 [10.7]
Mood disorder	49	3 [6.1]	NA***	44	0	NA***	26	0
Conduct disorder	49	9 [18.4]	NA***	44	3 [6.82]	NA***	26	0
Neurodevelopmental disorder	50	28 [56.0]	NA***	44	30 [68.2]	NA***	28	0

٭Not all participants were assessed for all psychiatric disorders; for one child in the deletion group and one child in the control group no psychiatric assessments were conducted, they were still included in the sample because cognitive measures, sociodemographic data and physical measures were obtained. None of the children in any group had an eating disorder, and one child with a deletion had a substance use disorder.

**For comparison with family controls.

***ORs could not be calculated because no family controls met criteria.

^a^With family ID in model: OR = 7.1 (1.2–44.1), *p* = 0.03.

^b^With family ID in model: OR = 1.5 (0.2–12.9), *p* = 0.71.

**Table 2:** The prevalence of psychiatric diagnoses in adults with 1q21 deletion or duplication and for adult controls.

Adults (*n* = 60)	Carrier status (*n*)							
	Deletion (*n* = 17)			Duplication (*n* = 11)			Controls (*n* = 32)	
Psychiatric diagnosis (DSM-IV/5)	Number assessed	With diagnosis [%]	OR (95% CI) and *p*-value*	Number assessed	With diagnosis [%]	OR (95% CI) and *p*-value*	Number assessed	With diagnosis [%]
Any psychiatric diagnosis	17	10 [58.8]	4.0 (1.2–15.0), 003^a^	11	8 [72.7]	6.0 (1.3–35.2), 0.03^e^	32	9 [28.1]
Anxiety disorder	17	4 [23.5]	1.8 (0.4–8.4), 0.46^b^	11	6 [54.6]	10.0 (1.9–71.2), 0.01^f^	32	5 [15.6]
Mood disorder	17	6 [35.3]	6.6 (1.4–40.1), 0.02^c^	11	6 [54.6]	8.3 (1.4–55.5), 0.02^g^	32	3 [9.4]
Substance use disorder	17	2 [11.8]	NA**	11	1 [9.1]	NA**	32	0
Neurodevelopmental disorder	17	4 [23.5]	2.2 (0.2–25.5), 0.49^d^	11	3 [27.3]	3.5 (0.4–41.6), 0.26	31	3 [9.7]

*For comparison with family controls.

**ORs could not be calculated because no family controls met criteria.

^a^With family ID in model: OR = 4.0 (1.1–14.2), *p* = 0.03.

^b^With family ID in model: OR = 1.8 (0.4–8.2), *p* = 0.46.

^c^With family ID in model: OR = 6.7 (0.5–85.4), *p* = 0.14.

^d^With family ID in model: OR = 2.2 (0.2–20.2), *p* = 0.49.

^e^With family ID in model: OR = 6.4 (0.92–44.7), *p* = 0.06.

^f^With family ID in model: OR = 10.0 (1.7–58.2), *p* = 0.01.

^g^With family ID in model: OR = 8.3 (1.4–49.4), *p* = 0.02.

#### Continuous variables

We conducted linear mixed-effect models for FSIQ, VIQ and PIQ, with group status (deletion vs duplication vs controls), age, and sex as fixed effects, and family as a random effect to take into account relatedness between 1q21.1 CNV carriers and controls. Post hoc Tukey tests were conducted to evaluate which groups differed for FSIQ, VIQ and PIQ. Statistical analysis of the difference in head circumferences between the participants with 1q21 deletion and duplication was conducted with t-test for independent groups, following conversion of raw scores to age-standardised *Z*-scores.

We transformed neuropsychiatric trait scores (only available for the Cardiff children) using the Tukey Ladder of Powers transformation to make the data fit the normal distribution as closely as possible^9^. We then standardised all transformed test scores into *Z-*scores using the mean and SD of the control group as reference—i.e., the difference in the individual’s score and the mean score for the control group was divided by the SD for the control group. We constructed these *Z-*scores so that a negative score denoted a worse outcome. We used linear mixed-effects models with test score as the outcome and carrier status (deletion, vs duplication vs controls), age and sex as fixed effects and family as a random effect.

## Results

### Psychopathology

#### Children

**Fig. 1:**
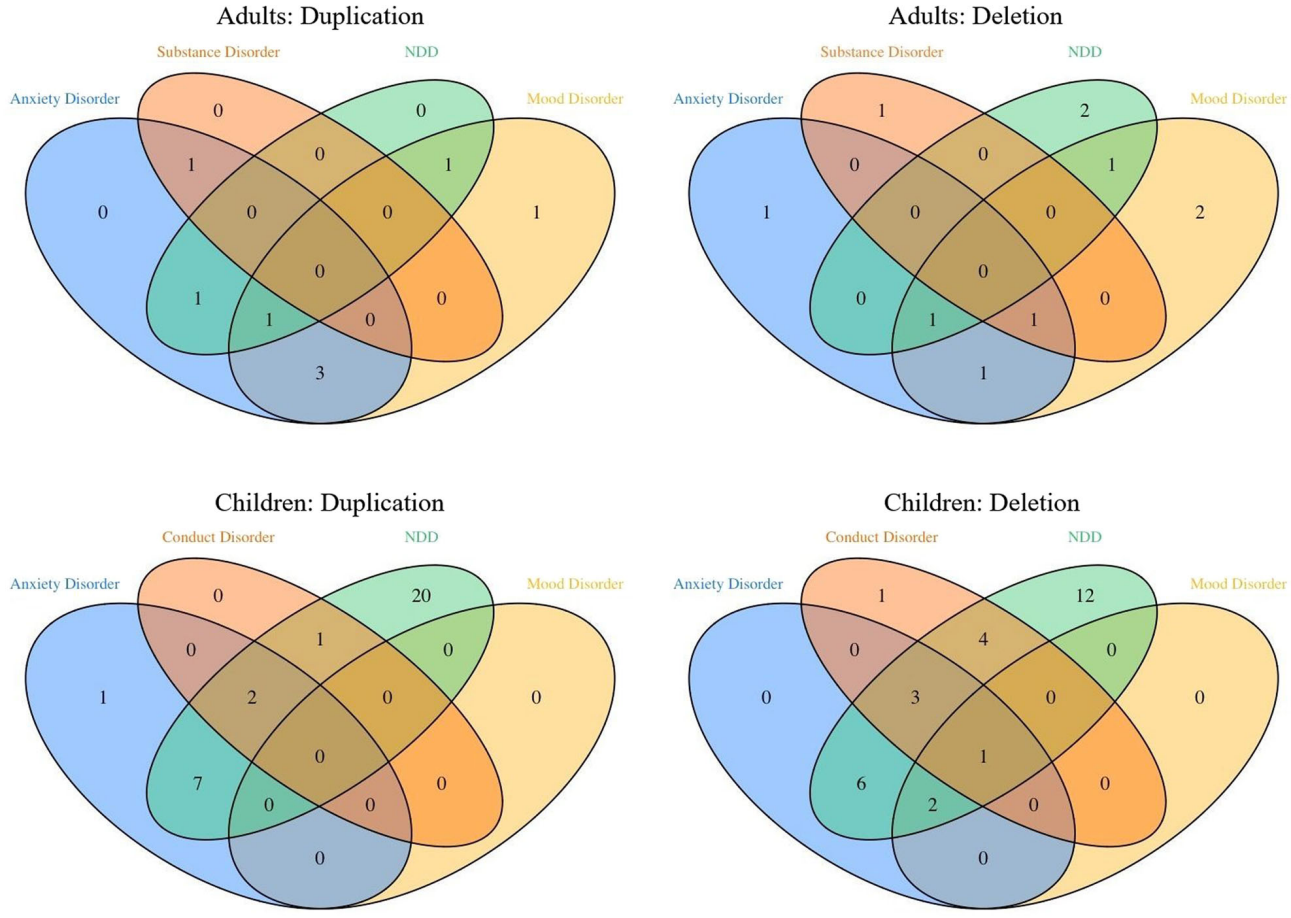
Diagnostic overlap of mental disorders. Venn diagrams showing number of main diagnoses (as well as multiple diagnoses: participants placed in the respective intersections) in children and adults with any psychiatric diagnosis.

The frequency of psychiatric disorders in the children with 1q21 CNVs is documented in Table 1 and Fig. 1. Both children with the deletion (OR = 7.11 (1.34–48.60), *p* = 0.03) and those with the duplication (OR = 12.24 (2.62–74.84), *p* = 0003) had higher frequencies of any psychiatric diagnosis compared to the family control sample. The prevalence of any neurodevelopmental disorder (NDD, including ASD, ADHD, ID, social communication disorder and specific learning disorder) was 56% in the deletion and 68% in the duplication carrier group as compared to 0% in the family controls.

The most prevalent NDD was ADHD both in children with the deletion (19/50 cases assessed for NDD; 38%) and children with the duplication (21/44 cases assessed for NDD; 47.7%) (Suppl. Table S3). The prevalence of any anxiety disorder was also higher in the deletion (25%) and duplication (23%) carrier groups as compared to the controls (11%), but these differences did not reach significance. Generalised anxiety disorder and specific phobia were the most common diagnoses in both the deletion and the duplication groups (Suppl. Table S4).

Children with 1q21.1 deletion had higher scores relative to control siblings for *ADHD traits* as measured by the CAPA (ADHD symptomatology, *z* = −2.85, *p* = 0.005) and the SDQ (Hyperactivity subscale, *z* = −2.10, *p* = 0.002), with higher scores indicating higher difficulty levels/psychopathology. Children with the 1q21.1 duplication had higher scores than control siblings for *ADHD traits* as measured by the CAPA (ADHD symptomatology, *z*− = −3.11, *p* = 0.005) and the SDQ (Hyperactivity subscale, *z* = −2.38, *p* = 0.003), and *traits of oppositional defiance* as measured by the CAPA (ODD symptomatology, *z* = −1.50, *p* = 0.006) and the SDQ (Conduct subscale, *z* = −2.08, *p* = 0.018). Comparing the two CNV groups, children with the deletion had higher levels of mood symptomatology (*z* difference = −0.56, *p* = 0.033), and ODD symptomatology (*z* difference = −1.01, *p* = 0.008) than children with the duplication. Full results of the CAPA scores are presented in the Supplementary Material 2.

#### Adult sample

The frequencies of mental disorders in the adults with 1q21 CNVs are documented in Table 2 and Fig. 1.

##### Deletion carriers versus family controls

Fifty-nine percent of the carriers had at least one psychiatric disorder compared to 28% in the control group (OR = 4.0, *p* = 0.03). The prevalence of any mood disorder was higher in the deletion carrier group as compared to the family controls (35% versus 9%, OR = 6.0, *p* = 0.02). No differences were found for any of the other diagnostic categories. The ORs were similar in the model with family ID included although the difference in mood disorder was no longer significant (from *p* = 0.02 to 0.14).

##### Duplication carriers versus family controls

Seventy-three percent of the carriers (versus 28% of the family controls) met criteria for any psychiatric disorder (OR = 6.0, *p* = 0.03). The prevalence of any mood disorder was higher in the duplication carrier group as compared to the family controls, (55% versus 9%, OR = 8.3, *p* = 0.02). The prevalence of any anxiety disorder was also higher in the duplication carrier group as compared to the family controls (55% versus 16%, OR = 10.0, *p* = 0.01). No differences were found for any of the other diagnostic categories. The ORs were similar in the model with family ID included although the difference in any psychiatric diagnosis now only reached trend level (*p* = 0.06).

### Seizure rates

Seven out of 36 children with deletions (19.4%) and 5/35 children with duplications (14.3%) had a history of unprovoked seizures. History of febrile seizures was reported for no child with deletion and 2/35 children with duplications (6%). Figures in adults were too low to allow for robust inferences but did not provide evidence for increased seizure rates (Suppl. Table S5).

### Cognitive function

IQ data is presented in Table 3. Full data on CANTAB subscores are presented in the Supplementary Material 2.

**Table 3:** Measures of intelligence for children and adults with 1q21.1 deletion or duplication and family controls.

	Mean VIQ* (SD) {range}	Mean PIQ* (SD) {range}	Mean FSIQ* (SD) {range}
*Children* (*n* = 80)			
Deletion (*n* = 33)	83.3** (17.9) {56–120}	85.3** (13.8) {59–114}	82.4** (15.4) {58–112}
Duplication (*n* = 30)	83.8** (19.0) {49–123}	90.3** (17.6) {57–126}	86.0** (18.3) {53–128}
Controls (*n* = 17)	97.6 (12.3) {75–115}	102.5 (9.0) {86–122}	99.9 (10.6) {83–121}
*Adults* (*n* = 44)			
Deletion (*n* = 11)	87.8** (18.8) {63–113}	95.7** (12.1) {73–117}	90.6** (15.4) {70–116}
Duplication (*n* = 9)	95.6 (13.5) {73–111}	106.0 (14.0) {82–125}	100.8 (12.6) {81–115}
Controls (*n* = 24)	106.9 (14.8) {78–128}	111.7 (12.9) {89–140}	111.5 (12.9) {83–134}

*Significant group difference (*F*-test) at *p* < 0.05.

**Significantly lower than in controls (Tukey post hoc tests) at *p* < 0.05. For further details see ‘Results’ section.

#### Children

The three groups differed for FSIQ (*F* = 7.01, df = 2, *p* = 0.002), PIQ (*F* = 7.62, df = 2, *p* < 0.001) and VIQ (*F* = 4.37, df = 2, *p* = 0.016). Post hoc tests (Tukey) indicated that deletion carriers had a lower FSIQ (*p* = 0.002), PIQ (*p* < 0.001) and VIQ (*p* = 0.029) than controls and similarly that duplication carriers had lower FSIQs, PIQs and VIQs than controls (FSIQ: *p* = 0.025; PIQ: *p* = 0.035; VIQ: *p* = 0.033). There were no IQ differences between deletion and duplication carriers (FSIQ: *p* = 0.582; PIQ: *p* = 0.274; VIQ: *p* = 0.996).

Children with 1q21.1 deletion had deficits relative to control siblings in *spatial cognition* including spatial planning (*z* = −1.46, *p* = 0.011), and spatial working memory (*z* = −2.23, *p* = 0.009). Children with the 1q21.1 duplication also had deficits relative to control siblings in spatial working memory (*z* = −2.71, *p* = 0.001). Children with the deletion had greater deficits in sustained attention than children with the duplication (*z* difference = −0.62, *p* = 0.020).

#### Adults

The three groups differed for FSIQ (*F* = 9.18, df = 2, *p* < 0.001), PIQ (*F* = 5.64, df = 2, *p* = 0.007) and VIQ (*F* = 5.77, df = 2, *p* = 0.006). Post hoc tests (Tukey) indicated that deletion carriers had a lower FSIQ than controls (*p* = 0.002), PIQ (*p* = 0.014) and VIQ (*p* = 0.025) but that there were no significant differences between duplication carriers and controls (FSIQ: *p* = 0.168; PIQ: *p* = 0.590; VIQ: *p* = 0.223). Moreover, there were no IQ differences between deletion and duplication carriers (FSIQ: *p* = 0.428; PIQ: *p* = 0.323; VIQ: *p* = 0.747).

### Head circumference

**Fig. 2:**
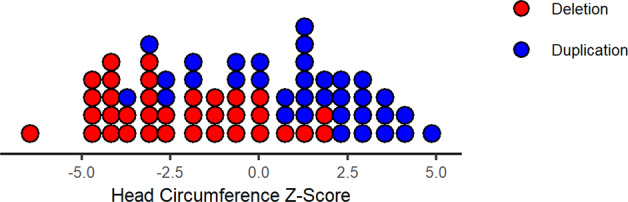
Distribution of head circumference. Age-normalised *Z*-scores for head circumference are plotted for 35 participants with 1q21 deletion (27 children and eight adults, age range 2–51) and 34 participants with 1q21 duplication (25 children and nine adults, age range 1–68).

Age-normalised head circumference data were available for 35 deletion and 34 duplication carriers (Fig. 2). Standardised head circumference was markedly lower for deletion vs. duplication carriers (*t* = −6.48 [95% CI = −4.48−2.37], df = 66.4, *p*-value = 1.3 × 10^−8^).

## Discussion

This is the largest and most detailed study to date documenting the psychiatric and cognitive phenotype associated with copy number variation at the 1q21 chromosomal locus. We found a clear association of deletion and duplication of the critical region of chromosome 1q21 with psychopathology both in children ascertained through clinical genetics services and an adult group that was mainly composed of parents of probands. As in previous studies, based on smaller samples, the main diagnostic groups of neurodevelopmental disorders were ADHD and ASD. Association of both the deletion and the duplication with ADHD- and ASD-related psychopathology was also revealed through quantitative measures (CAPA and SDQ) in the Cardiff children sample. However, the high prevalence of anxiety disorders in both deletion and duplication carriers (previously only reported for those with the deletion)^9^ and the high prevalence of mood and anxiety disorders in the high-functioning adult group are novel findings. These findings and the recent report of high prevalence of depression in participants of a population cohort (UK Biobank) with 1q21 duplication^32^ underline the association of the 1q21 locus with psychopathology beyond the classical neurodevelopmental disorders.

We found no evidence for preponderance of certain psychiatric syndromes in the deletion or duplication carriers, which some of the previous, smaller studies had suggested, in line with other studies highlighting commonalities across CNV dosage^33^. An implication of comparisons between carriers of deletions and duplications across multiple CNVs is that normal function seems to be supported by normal dosage of genes/proteins in the region, and deviations in both directions can be damaging. Furthermore, we found no evidence of psychiatric mirror phenotypes such as increased vs. reduced risk, which has been discussed for another CNV locus (22q11.2 deletion vs. duplication)^34^.

Similarly, seizure frequencies were high in both children with deletions and duplications. This represents an understudied topic in CNV research: however, a recent study has indicated that the frequency of seizures in young people with 22q11.2 deletion syndrome may be higher than suspected^35^, indicating that individuals with certain CNVs may need to be closely monitored and particularly under certain circumstances (e.g., fever). The one mirror phenotype of head size associated with this locus^6^ was confirmed in our study. We found the deletion was associated with smaller and the duplication with larger head circumference.

We initially hypothesised that both 1q21.1 deletions and duplications would result in cognitive impairment; however, the cognitive abilities in our study varied widely between the age-categories. Although each carrier group (except for the adults with a duplication) displayed significantly lower levels of cognitive ability than controls, the mean scores in the adult carriers were in the normal range, reflecting the notion that the child and adult carriers recruited to this study may fall in different parts of the 1q21.1 functional spectrum based upon method of ascertainment.

Like other neurodevelopmental CNVs, including 22q11.2 deletion syndrome^36^, the effect of 1q21 deletion and duplication on psychopathology is pleiotropic. This suggests that pathogenic CNVs are associated with cognitive impairment and psychological dysfunction^9^, perhaps through alterations in brain development^37^, rather than a specific psychiatric syndrome. Although the spectrum of phenotypes includes intellectual disability, and both 1q21 deletion and duplication are associated with reductions in mean IQ and educational attainment^4^, the present study shows that psychopathology in CNV carriers at this locus is not confined to those with a low IQ. Even the adult group, with a mean full-scale IQ in the average range, had high prevalence of psychopathology, mainly anxiety and mood disorders. Beyond this dissociation of intellectual impairment and psychopathology, our study reveals two subgroups of carriers of CNVs at 1q21: an intellectually impaired group that is referred to clinical genetics services in childhood because of developmental delay and/or physical abnormalities and has a high prevalence of neurodevelopmental disorders, but also of anxiety disorders, and a group with only subtle intellectual/educational impairment (but high prevalence of anxiety/mood disorders) which is generally not recognised unless they are referred for genetic testing because of their children. Similar patterns likely exist for other neurodevelopmental risk factors with variable penetrance, even those associated with recognised clinical syndromes such as 22q11.2 deletion. The mechanisms underlying this variable penetrance of pathogenic neurodevelopmental CNVs are still poorly understood and likely include both genetic (oligogenic and polygenic)^38^ and environmental modifiers. Large and multigenerational cohorts of CNV carriers are particularly suited to the study of modifying effects because they allow for the comparison of probands and their (generally less severely affected) parents and extended family members.

The experience of caring for a mentally ill child may contribute to a higher prevalence of stress-related disorders in parents. However, a recent large-scale study into the (sub)clinical symptoms of parents of children and adolescents with psychopathology found lower frequencies (depressive symptoms: 12.8% for fathers, 14.6% for mothers; anxiety: 6 and 7.2%^39^) than the present study, which suggests a genetic contribution to the psychopathology of the adult carriers. In addition to the importance for the integrated clinical care for families affected by CNVs at 1q21, these findings also have implications for translational neuroscience, highlighting the need to model the anxious phenotype in animal models of this locus and to identify the responsible genes.

The 1q21 critical region contains several genes that are expressed in the brain, including Hydrocephalus-inducing homologue 2 (*HYDIN2*) and three Notch homologue 2 N-terminal-like (*NOTCH2NL*) genes. It has recently been proposed that *NOTCH2NL* is crucial for the effects on cortical development^40^. Another proposed genetic mechanism for the effects on head/brain size is related to the Olduvai protein domain (formerly called DUF1220 domain). Sequences coding for this protein domain (average amino acid length ~65) are predominantly found in the 1q21 locus, primarily clustered in Neuroblastoma breakpoint family (NBPF) genes in close proximity to the *NOTCH2NL* genes^41^. The human lineage-specific increase in copy number of sequences coding for these domains has been linked with the anthropoid brain expansion^42^, and individual differences in copy number were associated with brain size in people with 1q21 deletion^43^. Because of the SNP array used, this study focussed on large CNV’s present at this locus and further studies, including sequencing and high resolution CNV analysis of large cohorts, will be necessary for a full elucidation of these genotype-phenotype relationships.

### Limitations

The collaborations reported in this study were established following the assessment of participants and hence there is some discrepancy in the assessment measures used by each research centre. Another limitation is the relatively small number of family controls, particularly in the children group. We had to use the same control sample for both deletion and duplication carriers, which resulted in difficulties controlling effects for familiality.

### Implications

The results of the present study support the need for more extensive genetic testing in psychiatry because it is likely that a considerable number of users of mental health services carry an undiagnosed genetic variant. A recent study in patient groups with intellectual disability and comorbid mental disorder identified recognised pathogenic CNVs in ~10%^44^. If genetic testing were to be offered routinely for this patient population in mental health services, this would not only inform their clinical care but also provide much-needed information about the frequency and severity of neurobehavioral challenges associated with CNVs.

## Supplementary information

Supplementary tables, figures and methods

Supplementary results
