# Correction: The psychiatric phenotypes of 1q21 distal deletion and duplication

**DOI:** 10.1038/s41398-021-01296-9

**Published:** 2021-06-18

**Authors:** Stefanie C. Linden, Cameron J. Watson, Jacqueline Smith, Samuel J. R. A. Chawner, Thomas M. Lancaster, Ffion Evans, Nigel Williams, David Skuse, F. Lucy Raymond, Jeremy Hall, Michael J. Owen, David E. J. Linden, LeeAnne Green-Snyder, Wendy K. Chung, Anne M. Maillard, Sébastien Jacquemont, Marianne B. M. van den Bree

**Affiliations:** 1 Department of Health, Ethics and Society, Care and Public Health Research Institute (CAPHRI), Faculty of Health, Medicine and Life Sciences, Maastricht University, Maastricht, The Netherlands; 2 Division of Psychological Medicine and Clinical Neurosciences, Medical Research Council Centre for Neuropsychiatric Genetics and Genomics, Cardiff University, Cardiff, UK; 3 Preventive Neurology Unit, Wolfson Institute of Preventive Medicine, Queen Mary University of London, London, UK; 4 School of Psychology, University of Bath, Bath, UK; 5 Behavioural and Brain Sciences Unit Institute of Child Health, University College London, London, UK; 6 Cambridge Institute for Medical Research, University of Cambridge, Cambridge, UK; 7 Department of Psychiatry and Neuropsychology, School for Mental Health and Neuroscience, Faculty of Health, Medicine and Live Sciences, Maastricht University, Maastricht, The Netherlands; 8 Simons Foundation, New York, NY USA; 9 Departments of Pediatrics and Medicine, Columbia University, New York, NY USA; 10 Service des Troubles du Spectre de l’Autisme et apparentés, Centre Hospitalier Universitaire Vaudois, University of Lausanne, Lausanne, Switzerland; 11 Service de Génétique Médicale, Centre Hospitalier Universitaire Vaudois, Lausanne, Switzerland

**Keywords:** Clinical genetics, Psychiatric disorders

Correction to: *Translational Psychiatry*

10.1038/s41398-021-01226-9 published online 4 February 2021

The original version of this article unfortunately contained a mistake in the ‘Discussion’ section. At the end of the first paragraph, the word ‘deletion’ should be replaced with ‘duplication’. The correct sentence is given below. The authors apologize for the mistake. The original article was revised.

‘These findings and the recent report of high prevalence of depression in participants of a population cohort (UK Biobank) with 1q21 duplication underline the association of the 1q21 locus with psychopathology beyond the classical neurodevelopmental disorders’.

