# Supplementary material for: The psychiatric phenotypes of 1q21 distal deletion and duplication
Source: Transl Psychiatry. 2021 Feb 4;11:105. doi: 10.1038/s41398-021-01226-9 (PMC7862693; doi:10.1038/s41398-021-01226-9)
Supplement: Supplementary file 1 — Supplementary tables, figures and methods [file 41398_2021_1226_MOESM1_ESM.docx]

SUPPLEMENTARY TABLES

Table S1: Description of 1q21.1 carrier groups

|  | **Deletions** | | | **Duplications** | | |
| --- | --- | --- | --- | --- | --- | --- |
| **Cohort by carrier group** | **Sample size** | **Male/Female** | **Age, mean (SD)** | **Sample size** | **Male/Female** | **Age, mean (SD)** |
| **CHILDREN** | **51** | **30/21** | **8.3 (3.9)** | **44** | **27/17** | **8.4 (3.7)** |
| **UK** |  |  |  |  |  |  |
| Probands | 18 | 14/4 | 9.44 (2.77) | 22 | 12/10 | 8.82  (2.9) |
| Familial carriers | 4 | 1/3 | 10.25 (1.50) | 5 | 3/2 | 10.20 (4.3) |
| **Lausanne** |  |  |  |  |  |  |
| Probands | 5 | 1/4 | 7.40 (3.78) | 1 | 1/0 | 15.00 (0.00) |
| Familial carriers | 1 | 0/1 | 13.00 (0.00) |  |  |  |
| **Simons VIP** |  |  |  |  |  |  |
| Probands | 21 | 13/8 | 6.71 (4.562) | 11 | 7/4 | 7.18 (4.4) |
| Familial carriers | 2 | 1/1 | 10.00 (2.828) | 3 | 2/1 | 6.00 (4.4) |
| Status unknown |  |  |  | 2 | 2/0 | 7.00 (0.000) |
|  | | | | |  |  |
| **ADULTS** | **17** | **7/10** | **34.7 (8.9)** | **11** | **3/8** | **46.5 (18)** |
| **UK** |  |  |  |  |  |  |
| Probands | 2 | 1/1 | 24.50 (7.778) | 1 | 0/1 | 68.0(0.000) |
| Familial carriers | 4 | 3/1 | 40.50 (11.030) | 4 | 1/3 | 37.5 (10.847) |
| **Lausanne** |  |  |  |  |  |  |
| Probands |  |  |  |  |  |  |
| Familial carriers | 4 | 1/3 | 37.00 (2.160) |  |  |  |
| Status unknown |  |  |  | 1 | 0/1 | 65.0 (0.000) |
| **Simons VIP** |  |  |  |  |  |  |
| Probands | 1 | 0/1 | 35.00 (0.000) | 1 | 0/1 | 20.00(0.000) |
| Familial carriers | 5 | 2/3 | 31.20 (9.654) | 2 | 2/0 | 44.50 (10.607) |
| Status unknown | 1 | 0/1 | 39.00 (0.000) | 2 | 0/2 | 59.50 (24.749) |

Familial carriers were defined as relatives who carry the CNV identified through family cascade testing but who were not referred for a neurodevelopmental disorder.

Table S2: Sociodemographic Statistical Comparison

No significant age or gender differences with controls were observed in either the child or adult cohort (p values are provided for the appropriate tests: two-tailed independent samples t tests for age; chi square tests for gender).

|  | **Del vs. controls (p)** | **Dup vs. controls (p)** |
| --- | --- | --- |
| **Age: Children** | 0.390 | 0.313 |
| **Gender: Children** | 0.602 | 0.485 |
| **Age: Adults** | 0.088 | 0.063 |
| **Gender: Adults** | 0.512 | 0.241 |

Table S3: Breakdown of common diagnoses for children (NDD)

| **Diagnosis** | **Breakdown of NDD in children with 1q21.1 deletion*** | **Breakdown of NDD in children with 1q21.1 duplication**** |
| --- | --- | --- |
| Autism spectrum disorder/ Autistic disorder | 4/28 (14.4%) | 9/30 (30%) |
| ADHD | 19/28 (67.7%) | 21/30 (70%) |
| Intellectual disability | 7/28 (25%) | 5/30 (16.7%) |
| Specific learning disorder | 2/28 (7.1%) | 6/30 (20%) |
| Social communication disorder | 1/28 (3.6%) | 1/30 (3.3%) |

*A total of 18 children with 1q21.1 deletion had a NDD diagnosis

**A total of 30 children with 1q21.1 duplication had a NDD diagnosis

Table S4: Breakdown of common diagnoses for children (anxiety disorders)

| **Diagnosis** | **Breakdown of anxiety disorders in children with 1q21.1 deletion** | **Breakdown of anxiety disorders in children with 1q21.1 duplication** |
| --- | --- | --- |
| Separation anxiety disorder | 1/12 (8.3%) | 0/10 |
| Generalised anxiety disorder | 6/12 (50%) | 4/10 (40%) |
| Specific phobias | 4/ 12 (33.3%) | 4/10 (40%) |
| Social phobia | 2/ 12(16.7%) | 3/10 (30%) |
| PTSD | 1/12 (8.3%) | 1/10 (10%) |

Table S5: Seizure frequency in 1q21 deletion and duplication carriers (history of any seizure).

|  | Children (%) | Adults (%) |
| --- | --- | --- |
| Deletion - Unprovoked | 7/ 36 (19%) | 1/12 (8%) |
| Deletion – Febrile | 0 | 0 |
| Duplication - Unprovoked | 5/35 (14%) | 0 |
| Duplication - Febrile | 2/35 (6%) | 1/10 (10%) |

Note: We did not have seizure history information for 15 children with deletion and 9 children with duplication and for 5 adults with deletion and one adult with duplication. All control children and adults had unknown seizure history.

SUPPLEMENTARY FIGURES

Figure S1: Estimated breakpoints (GRC37/hg19) of each deletion relative to the flanking segmental duplications at 1q21.

Figure S2: Estimated breakpoints (GRC37/hg19) of each duplication relative to the flanking segmental duplications at 1q21.

SUPPLEMENTARY METHODS

**IMAGINE-ID Consortium Membership**

^1^ School of Clinical Medicine, University of Cambridge, Cambridge Biomedical Campus, Cambridge, UK

^2^ Medical Research Council Centre for Neuropsychiatric Genetics and Genomics, Division of Psychological Medicine and Clinical Neurosciences, and Neuroscience and Mental Health Research Institute, Cardiff University, Cardiff, UK.

^3^ NIHR BRC Great Ormond Street Institute of Child Health, University College London, London, UK

^4^ Unique – The Rare Chromosome Disorder Support Group, London, UK.

| **Title (if applicable)** | **First Name** | **Surname** | **Institution** |
| --- | --- | --- | --- |
| Dr | Kate | Baker | 1 |
|  | Eleanor | Dewhurst | 1 |
|  | Amy | Lafont | 1 |
| Professor | F Lucy | Raymond | 1 |
|  | Terry | Shirley | 1 |
|  | Hayley | Tilley | 1 |
|  | Husne | Timur | 1 |
|  | Catherine | Titterton | 1 |
|  | Neil | Walker | 1 |
|  | Sarah | Wallwork | 1 |
|  | Francesca | Wicks | 1 |
| Dr | Zheng | Ye | 1 |
|  | Marie | Erwood | 1 |
|  | Sophie | Andrews | 2 |
|  | Philippa | Birch | 2 |
|  | Samantha | Bowen | 2 |
|  | Karen | Bradley | 2 |
|  | Aimee | Challenger | 2 |
| Dr | Samuel | Chawner | 2 |
| Dr | Andrew | Cuthbert | 2 |
| Professor | Jeremy | Hall | 2 |
| Professor | Peter | Holmans | 2 |
|  | Sarah | Law | 2 |
|  | Nicola | Lewis | 2 |
|  | Sinead | Morrison | 2 |
|  | Hayley | Moss | 2 |
| Professor Sir | Michael | Owen | 2 |
|  | Sinead | Ray | 2 |
|  | Matthew | Sopp | 2 |
|  | Molly | Tong | 2 |
| Professor | Marianne | van den Bree | 2 |
|  | Nadia | Coscini | 3 |
|  | Sarah | Davies | 3 |
|  | Spiros | Denaxas | 3 |
|  | Hayley | Denyer | 3 |
|  | Nasrtullah | Fatih | 3 |
|  | Manoj | Juj | 3 |
|  | Ellie | Kerry | 3 |
|  | Anna | Lucock | 3 |
| Dr | William | Mandy | 3 |
|  | Frida | Printzlau | 3 |
| Professor | David | Skuse | 3 |
| Dr | Ramya | Srinivasan | 3 |
| Dr | Susan | Walker | 3 |
|  | Alice | Watkins | 3 |
| Dr | Jeanne | Wolstencroft | 3 |
| Dr | Beverly | Searle | 4 |
| Dr | Anna | Pelling | 4 |

IMAGINE-ID Clinical Collaborators

| **Title (if applicable)** | **First Name** | **Surname** | **Shorthand Institution** | **Genetics service** | **Hospital Trust** |
| --- | --- | --- | --- | --- | --- |
| Dr | John | Dean | Aberdeen | Aberdeen Royal Infirmary Genetics Service | NHS GRAMPIAN |
| Dr | Lisa | Robertson | Aberdeen | Aberdeen Royal Infirmary Genetics Service | NHS GRAMPIAN |
| Dr | Denise | Williams | Birmingham | West Midlands Regional Genetics Service | BIRMINGHAM WOMEN'S NHS FOUNDATION TRUST |
| Dr | Alan | Donaldson | Bristol | Bristol Clinical Genetics Service | UNIVERSITY HOSPITALS BRISTOL NHS FOUNDATION TRUST |
| Professor | Lucy | Raymond | Cambridge | East Anglian Medical Genetics Service | CAMBRIDGE UNIVERSITY HOSPITALS NHS FOUNDATION TRUST |
| Dr | Annie | Procter | Cardiff | All Wales Regional Genetics Service | CARDIFF AND VALE UNIVERSITY LHB |
| Dr | Jonathan | Berg | Dundee | Ninewells Hospital Dundee Genetics Service | NHS TAYSIDE |
|  | Yanick | Crow | Edinburgh | Western General Hospital Edinburgh Genetics Service | NHS LOTHIAN |
| Professor | Anne | Lampe | Edinburgh | Western General Hospital Edinburgh Genetics Service | NHS LOTHIAN |
| Dr | Julia | Rankin | Exeter | Peninsula Genetics Service | ROYAL DEVON AND EXETER NHS FOUNDATION TRUST |
| Dr | Shelagh | Joss | Glasgow | Glasgow Genetics Centre | NHS GREATER GLASGOW & CLYDE |
| Professor | Lyn | Chitty | GOSH | London North East Thames Regional Genetics Service - Clinical Genetics | GREAT ORMOND STREET HOSPITAL FOR CHILDREN NHS FOUNDATION TRUST |
| Professor | Frances | Flinter | Guy's | London Guy's Hospital Genetic Centre | GUY'S AND ST THOMAS' NHS FOUNDATION TRUST |
| Dr | Muriel | Holder | Guy's | London Guy's Hospital Genetic Centre | GUY'S AND ST THOMAS' NHS FOUNDATION TRUST |
| Dr | Alison | Kraus | Leeds | Yorkshire Regional Genetics Service - Clinical Genetics | LEEDS TEACHING HOSPITALS NHS TRUST |
| Dr | Julian | Barwell | Leicester | Leicestershire Genetics Centre | UNIVERSITY HOSPITALS OF LEICESTER NHS TRUST |
| Dr | Pradeep | Vasudevan | Leicester | Leicestershire Genetics Centre | UNIVERSITY HOSPITALS OF LEICESTER NHS TRUST |
| Dr | Astrid | Weber | Liverpool | Cheshire & Merseyside Regional Genetic Service | LIVERPOOL WOMEN'S NHS FOUNDATION TRUST |
| Dr | William | Newman | Manchester | Manchester Centre for Genomic Medicine | CENTRAL MANCHESTER UNIVERSITY HOSPITALS NHS FOUNDATION TRUST |
| Dr | Miranda | Splitt | Newcastle | Northern Genetics Service | THE NEWCASTLE UPON TYNE HOSPITALS NHS FOUNDATION TRUST |
| Dr | Virginia | Clowes | North West Thames | London North West Thames Regional Genetics Service | NORTH WEST LONDON HOSPITALS NHS TRUST |
| Dr | Fleur | van Dijk | North West Thames | London North West Thames Regional Genetics Service | NORTH WEST LONDON HOSPITALS NHS TRUST |
| Dr | Rachel | Harrison | Nottingham | Nottingham Regional Genetics Service | NOTTINGHAM UNIVERSITY HOSPITALS NHS TRUST |
| Dr | Usha | Kini | Oxford | Oxford Genetics Service | OXFORD UNIVERSITY HOSPITALS NHS TRUST |
| Dr | Oliver | Quarrell | Sheffield | Sheffield Genetic Services | SHEFFIELD CHILDREN'S NHS FOUNDATION TRUST |
| Dr | Diana | Baralle | Southampton | Wessex Clinical Genetics Service | UNIVERSITY HOSPITAL SOUTHAMPTON NHS FOUNDATION TRUST |
| Dr | Sahar | Mansour | St George's | London South West Thames Regional Genetics Service | ST GEORGE'S HEALTHCARE NHS FOUNDATION TRUST |
