# Supplementary material for: The psychiatric phenotypes of 1q21 distal deletion and duplication
Source: Transl Psychiatry. 2021 Feb 4;11:105. doi: 10.1038/s41398-021-01226-9 (PMC7862693; doi:10.1038/s41398-021-01226-9)
Supplement: Supplementary file 2 — Supplementary results [file 41398_2021_1226_MOESM2_ESM.pdf]

Trait	Measure	1q21.1 duplication			1q21.1 deletion			Controls			Post hoc contrasts p-values		
		N	zscore	sd	N	zscore	sd	N	zscore	sd	Del vs Dup	Dup vs control	Del vs control
Reaction time	CANTAB	27	-0.29	0.79	22	-0.44	0.91	9	0.00	1.00	0.703202759	0.493679831	0.197555909
Sustained attention	CANTAB	27	-0.14	0.87	22	-0.76	0.97	9	0.00	1.00	0.019615121	0.986074783	0.059584813
Spatial planning	CANTAB	27	-1.46	1.35	22	-1.03	1.44	9	0.00	1.00	0.549620954	0.01136307	0.102590846
Spatial working memory	CANTAB	26	-2.23	2.18	21	-2.71	2.08	9	0.00	1.00	0.669006274	0.008597894	0.001055182
Set shifting	WCST	20	-0.18	1.28	21	-0.61	1.23	5	0.00	1.00	0.519742935	0.95682942	0.586774238
Total psychiatric symptomatology	CAPA	27	-0.63	1.04	20	-1.22	1.22	6	0.00	1.00	0.081656343	0.616705217	0.059577838
ADHD symptomatology	CAPA	27	-2.85	2.27	20	-3.11	2.48	6	0.00	1.00	0.850148526	0.005033093	0.004470863
Anxiety symptomatology	CAPA	27	-0.21	1.07	20	-0.38	1.21	6	0.00	1.00	0.603103029	0.994531772	0.841896991
Mood symptomatology	CAPA	27	0.10	0.62	20	-0.46	0.89	6	0.00	1.00	0.033125798	0.946999001	0.425141887
OCD symptomatology	CAPA	27	-1.20	5.44	20	-0.81	5.36	6	0.00	1.00	0.999106324	0.928348015	0.933266478
ODD symptomatology	CAPA	27	-0.49	1.06	20	-1.50	1.15	6	0.00	1.00	0.007936424	0.387899031	0.006147475
Psychotic expereinces	CAPA	25	0.56	0.98	21	-2.17	13.37	6	0.00	1.00	0.264220585	1	0.264220585
Sleep symptomatology	CAPA	27	0.63	0.94	20	0.24	0.91	6	0.00	1.00	0.218243446	0.148624436	0.739099359
Motor functioning	DCDQ	25	-0.48	0.52	13	-0.59	0.46	4	0.00	1.00	0.884330919	0.216877824	0.138143514
Autism traits	SCQ	25	-1.06	1.02	14	-1.08	0.85	4	0.00	1.00	0.999860215	0.092411702	0.108561434
Total SDQ score	SDQ	25	-1.05	0.90	14	-1.67	1.32	5	0.00	1.00	0.172143697	0.005068842	0.000612211
Conduct problems	SDQ	25	-1.04	1.40	14	-2.08	1.67	5	0.00	1.00	0.103639658	0.29792513	0.018328207
Emotional problems	SDQ	25	0.11	0.62	14	-0.28	0.65	5	0.00	1.00	0.189143178	0.877029006	0.756146723
Hyperactivity problems	SDQ	25	-2.10	1.41	14	-2.38	1.55	5	0.00	1.00	0.764997601	0.002413011	0.002893285
Peer problems	SDQ	25	-0.80	1.21	14	-0.78	1.29	5	0.00	1.00	0.995463443	0.151521354	0.195313079
Prosocial problems	SDQ	25	-0.39	0.85	14	-0.34	0.96	5	0.00	1.00	0.995295599	0.377566341	0.485945369
